# Synthesis of 2-substituted 9-oxa-guanines {5-aminooxazolo[5,4-*d*]pyrimidin-7(6*H*)-ones} and 9-oxa-2-thio-xanthines {5-mercaptooxazolo[5,4-*d*]pyrimidin-7(6*H*)-ones}

**DOI:** 10.3762/bjoc.4.26

**Published:** 2008-07-25

**Authors:** Subrata Mandal, Wen Tai Li, Yan Bai, Jon D Robertus, Sean M Kerwin

**Affiliations:** 1 College of Pharmacy, 1 University Station, University of Texas, Austin, TX, 78712, USA; 2 Department of Biochemistry,1 University Station, University of Texas, Austin, TX, 78712, USA

**Keywords:** Annulation, Click Chemistry, Cyclization, Purine Analogs, Ricin

## Abstract

Oxazolo[5,4-*d*]pyrimidines can be considered as 9-oxa-purine analogs of naturally occurring nucleic acid bases. Interest in this ring system has increased due to recent reports of biologically active derivatives. In particular, 5-aminooxazolo[5,4-*d*]pyrimidine-7(6*H*)-ones (9-oxa-guanines) have been shown to inhibit ricin. The preparation of a series of 2-substituted 5-aminooxazolo[5,4-*d*]pyrimidin-7(6*H*)-ones and related 5-thio-oxazolo[5,4-*d*]pyrimidines is described, including analogs suitable for further elaboration employing “click” chemistry utilizing copper-catalyzed Huisgen 1,3-dipolar cycloadditions. Two of the compounds prepared were found to inhibit ricin with IC_50_ *ca.* 1–3 mM.

## Introduction

Oxazolo[5,4-*d*]pyrimidines have been reported to possess a variety of biological activities including kinase inhibition [1–2], adenosine receptor antagonism [3] and tumor growth inhibition [4]. In particular, 5-aminooxazolo[5,4-*d*]pyrimidin-7(6*H*)-ones [9-oxa-guanines] and related heterocycles have been shown to inhibit the ability of ricin to inactivate ribosomes [5]. Given these reports, and the expectation of further applications based upon similarity to naturally occurring nucleic acid bases, this heterocyclic ring system continues to generate interest.

**Figure 1:**
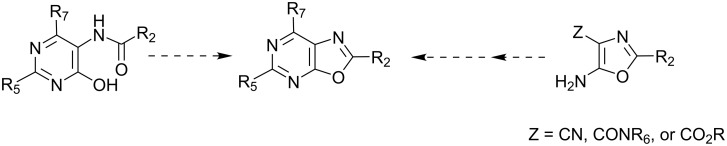
The two general synthetic approaches to oxazolo[5,4-*d*]pyrimidines.

Approaches to the oxazolo[5,4-*d*]pyrimidine ring system generally involve either cyclodehydration of an 5-(acylamino)-4-hydroxypyrimidine [6–10] or elaboration of a 4-cyano- or 4-(alkoxycarbonyl)-5-aminooxazole [11–15] (Figure 1), with only isolated reports of alternative routes [16–17]. However, few publications have described the preparation of 5-aminooxazolo[5,4-*d*]pyrimidin-7(6*H*)-ones [5,8,14] or 5-mercaptooxazolo[5,4-*d*]pyrimidin-7(6*H*)-ones [9-oxa-2-thio-xanthines] [7,15], and in both cases, the conditions do not appear to be amenable to the preparation of oxazolo[5,4-*d*]pyrimidines with variations at the 2-position, particularly those with additional functional groups or increased steric demand.

In pursuing 8-methyl-9-oxa-guanine [2-methyloxazolo[5,4-*d*]pyrimidin-7(6*H*)-one] as an initial lead in the design of inhibitors of the ribosome-inactivating protein ricin [5], we set out to prepare a series of 2-substituted 5-aminooxazolo[5,4-*d*]pyrimidin-7(6*H*)-ones and related 5-thio-oxazolo[5,4-*d*]pyrimidines. Here we report these studies, which have led to the preparation of analogs suitable for further elaboration, for example by “click” chemistry employing copper-catalyzed Huisgen 1,3-dipolar cycloadditions [18].

## Results and Discussion

**Figure 2:**
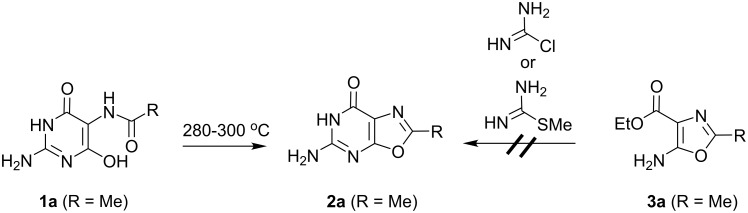
Thermal cyclodehydration route to 9-oxo-guanine.

Our previous route [5] to 8-methyl-9-oxa-guanine (**2a**) involved the thermal cyclodehydration of 5-(acetylamino)-2-amino-4,6-dihydroxypyrimidine (**1a**) [19] (Figure 2). Unfortunately, for other 5-acylamino analogs of **1a**, this route failed to afford the required oxazolo[5,4-*d*]pyrimidines, presumably due to decomposition of the product at the temperatures required for cyclodehydration. Other cyclodehydration conditions (POCl_3_, PPA) were also unsuccessful.

**Figure 3:**
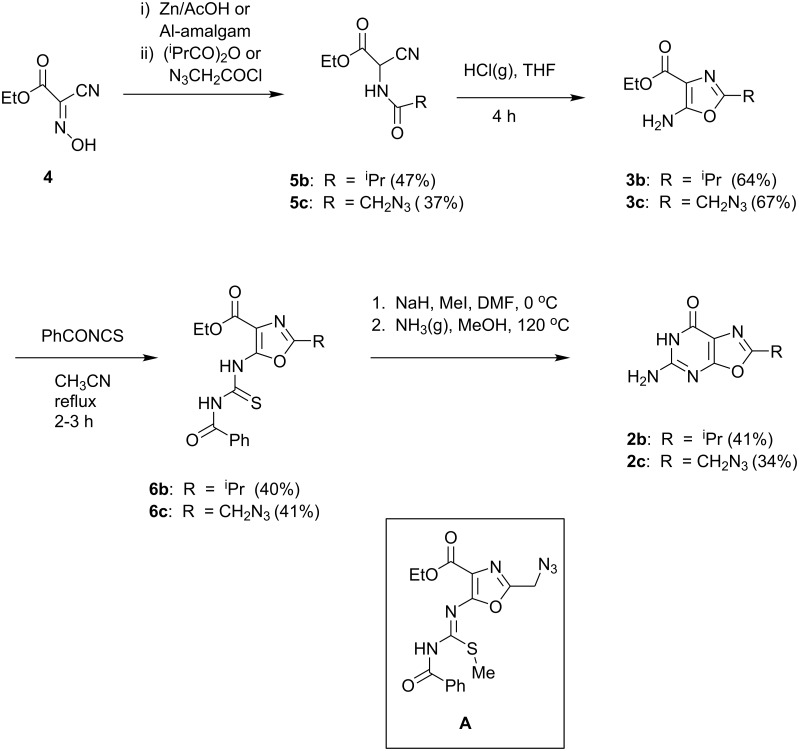
Preparation of 2-substituted 5-aminooxazolo[5,4-*d*]pyrimidin-7(6*H*)-ones.

An alternative route was sought in which the oxazole ring was formed first, followed by elaboration of the pyrimidine ring. Our previous work had demonstrated that in the case of ethyl 5-amino-2-methyloxazole-4-carboxylate **3a** [20], direct annulation with chloroformamidine in DMSO at 120 °C or with 2-methylisothiourea sulfate neat or in ethylene glycol at 170 °C did not afford the oxazolo[5,4-*d*]pyrimidine **2** (Figure 2). Thus, a stepwise annulation [15] strategy was explored for the preparation of analogs of **2** bearing different 2-positions substituents. The required 5-aminooxazoles **3b,c** were prepared from the (acylamino)cyanoacetates **5b,c**, which in turn were derived from ethyl cyanoglyoxylate oxime (**4**) by reduction followed by acylation by isobutyric anhydride or azidoacetyl chloride (Figure 3). Reaction of the aminooxazoles **3b,c** with benzoylisothiocyanate afforded the thioureas **6b,c**. Direct cyclization of **6b,c** to oxazolo[5,4-*d*]pyrimidines **2b,c** by treatment with ammonia in methanol was unsuccessful. However, thioureas **6b,c** were converted to the desired 2-substituted the oxazolo[5,4-*d*]pyrimidines **2b,c** by stepwise methylation followed by cyclization in the presence of ammonia in methanol. Although the intermediate products after methylation were not purified, in the case of **6c** the ^1^H NMR spectrum of the crude product following methylation retains the signals for the ethoxy group of **6c** and displays a new resonance for the *S*-methyl group at 2.0 ppm (see Supporting Information File 1), indicating that the intermediate is the *S*-methylisothiourea **A** (Figure 3). The mechanism involved in the conversion of intermediates such as **A** to **2** is not clear. It is unlikely that the 5-(methylthio)oxazolo[5,4-*d*]pyrimidine is an intermediate because treatment of 5-(methylthio)oxazolo[5,4-*d*]pyrimidine **8a** (see below) with ammonia in methanol fails to afford **2a**.

**Figure 4:**
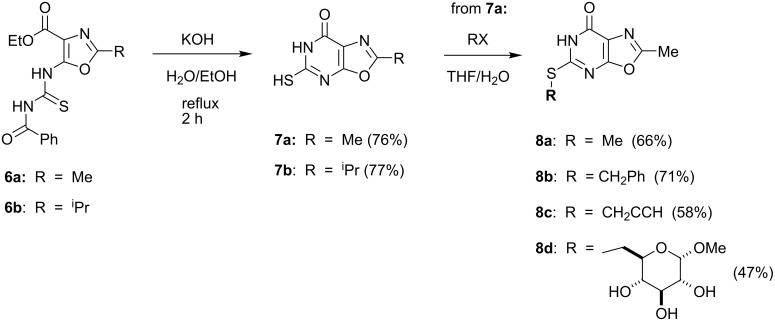
Preparation of 2-substituted 5-aminooxazolo[5,4-*d*]pyrimidin-7(6*H*)-ones and related thioethers.

The thiourea **6b** and its analog **6a**, which was prepared from aminooxazole **3a** in 51% yield, were also subjected to cyclization in the presence of ethanolic KOH to afford the 5-mercaptooxazolo[5,4-*d*]pyrimidin-7(6*H*)-ones **7a,b** (Figure 4). Further elaboration of **7a** by alkylation with methyl iodide, benzyl bromide, propargyl bromide, or methyl 6-*O*-(tolylsulfonyl)-α-D-glucopyranoside [21] afforded the thioethers **8a**–**d**, respectively. The structural assignment of **8a**–**d** as thioethers rest principally on their ^1^H and ^13^C NMR spectra, which contain resonances more commensurate with groups attached to sulfur (e.g., for **8a**: 2.2 ppm in ^1^H and 13 ppm in ^13^C NMR, respectively) than to nitrogen, as would be the case for 4- or 3-alkylated 5-mercaptooxazolo[5,4-*d*]pyrimidine-7(6*H*)-ones.

**Figure 5:**
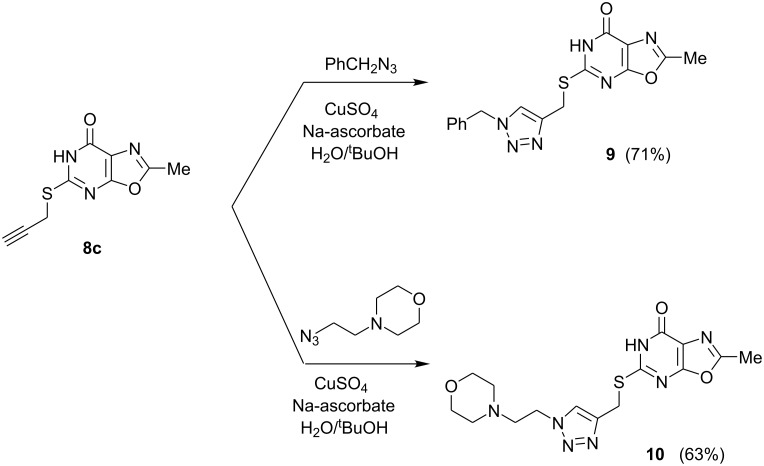
Click chemistry elaboration of a 5-(propargylthio)oxazolo[5,4-*d*]pyrimidine.

The thioether **8c** bearing a propargyl substituent was subjected to “click” chemistry coupling with benzyl azide or 2-morpholinoethyl azide in the presence of catalytic CuSO_4_ and sodium ascorbate to afford the triazoles **9** and **10**, respectively, in good yield (Figure 5). The copper-catalyzed Huisgen cycloaddition of terminal alkynes and alkyl azides favors formation of the 1,4-triazole regioisomers [22], and in the case of triazole **9**, this regiochemistry was confirmed by ^1^H NOE spectra (see Supporting Information File 1).

## Conclusion

In conclusion, routes to functionalized oxazolo[5,4-*d*]pyrimidines from 2-substituted 5-aminooxazole-4-carboxylic acid ethyl esters were developed, the key to which is the relatively mild conditions employed in the step-wise elaboration of the pyrimidine ring. Compounds **2b,c**, **7a,b**, **8a**–**d**, **9**, and **10** were evaluated for their ability to inhibit recombinant, catalytically active ricin A-chain (RTA) employing a modification of the previously reported assay [5]. Briefly, the synthesis of protein from endogenous globin mRNA by rabbit reticulocyte lysate was determined in the absence of RTA, in the presence of sufficient RTA to inhibit 90% of protein synthesis (*ca.* 10 pM), and in the presence of both RTA and increasing concentrations of the oxazolo[5,4-*d*]pyrimidines. The ability of these compounds to inhibit RTA and thereby rescue protein synthesis was determined. Compounds **2b** (IC_50_ = 2.8 mM) and **9** (IC_50_ = 1.6 mM), displayed some activity; whereas, none of the other compounds examined showed any significant RTA inhibitory activity.

## Supporting Information

File 1 This file includes full experimental details for all new compounds.

File 2 Copies of ^1^H and ^13^C NMR spectra of compounds **2b**,**c**; **3a**–**c**; **5b**,**c**; **6a**–**c**; **A**; **7a**,**b**; **8a**–**d**; **9**; and **10**.
