# Supplementary material for: Synthesis of 2-substituted 9-oxa-guanines {5-aminooxazolo[5,4-d]pyrimidin-7(6H)-ones} and 9-oxa-2-thio-xanthines {5-mercaptooxazolo[5,4-d]pyrimidin-7(6H)-ones}
Source: Beilstein J Org Chem. 2008 Jul 25;4:26. doi: 10.3762/bjoc.4.26 (PMC2511024; doi:10.3762/bjoc.4.26)
Supplement: File 1 — This file includes full experimental details for all new compounds. [file Beilstein_J_Org_Chem-04-26-s001.doc]

**Supporting Information: Experimental Details**

**Synthesis of 2-substituted 9-oxa-guanines {5-aminooxazolo[5,4-*d*]pyrimidin-7(6*H*)-ones} and 9-oxa-2-thio-xanthines {5-mercaptooxazolo[5,4-*d*]pyrimidin-7(6*H*)-ones}**

Subrata Mandal^1^, Wen Tai Li^1^, Yan Bai^2^, Jon D. Robertus^2^ and Sean M. Kerwin*^,1,2^

Address: ^1^College of Pharmacy, 1 University Station, University of Texas, Austin, TX, 78712, USA and ^2^Department of Biochemistry,1 University Station, University of Texas, Austin, TX, 78712, USA

Email: Sean M. Kerwin - [skerwin@mail.utexas.edu](mailto:skerwin@mail.utexas.edu)

* Corresponding author

**Experimental Section**

For anhydrous reactions, glassware was dried in an oven at 120 °C for at least 6 h. Reagents were purchased from commercial sources. All reaction solvents were distilled before use, and stored over activated 4 Å molecular sieves, unless otherwise indicated. Anhydrous tetrahydrofuran (THF) was obtained by distillation from sodium/ benzophenone under argon. Anhydrous CH_2_Cl_2_ was obtained by distillation from CaH_2_. NMR spectra were obtained on spectrometers operating at 300 or 400 MHz. All NMR chemical shifts are reported in parts per million (ppm), and coupling constants (*J*) are given in hertz (Hz). The splitting pattern abbreviations are described as follows: s, singlet; d, doublet; t, triplet; q, quartet; sept, septuplet; br, broad; m, unresolved multiplet due to the field strength of the instrument; and dd, doublet of doublet. High-resolution mass spectrometry (HRMS) measurements are valid to 5 ppm. Flash chromatography was carried out using Sorbent technologies (40-63 μm, 60 Å) silica gel. R_f_ values are reported for analytical thin-layer chromatography (TLC) performed on Waterman silica gel UV_254_ (250 μm) using the mobile phase indicated with visualizatioin by 254 nm light, 2% potassium permanganate in 4% aqueous sodium bicarbonate or 5% w/v phosphomolybdic acid in 95% EtOH.

**5-Amino-2-isopropyloxazolo[5,4-*d*]pyrimidin-7(6*H*)-one (2b).** To a solution of **6b** (42.2 mg, 0.13 mmol) in DMF (7 mL) was added NaH (60% in mineral oil, 6 mg, 0.15 mmol) at 0 °C. The mixture was stirred for 5 min at 0 °C and CH_3_I (10 μL, 0.15 mmol) was added dropwise. After an additional 30 min at 0 °C, the reaction mixture was allowed to warm to room temperature, and the solvent was removed *in vacuo.* To the residue was added 4 mL of a 2.0 M NH_3_ solution in CH_3_OH (4 mL). The reaction mixture was heated in a sealed glass tube at 120 °C for 2 h. The reaction mixture was cooled to room temperature, and evaporated. The residue was purified by flash column chromatography (10% CH_3_OH in EtOAc) to afford the title compound **2b** as a white solid that was only sparingly soluble in MeOH, DMSO, or H_2_O (10.3 mg, 41%). Mp > 250 °C. ^1^H NMR (400 MHz, 0.5% NaOH in D_2_O) δ 3.03-2.92 (sept, J = 6.9 Hz, 1H), 1.17 (d, *J* = 6.9 Hz, 6H). ^13^C NMR (100 MHz, 0.5% NaOH in D_2_O) δ 166.8, 165.2, 163.8, 159.8, 111.4, 29.4, 20.6. IR (KBr) 3381, 1678, 1583, 1252 cm^-1^. MS (CI) m/z 195 (MH^+^). HRMS calcd for C_8_H_11_N_4_O_2_ (MH^+^) 195.0882, found 195.0884

**5-Amino-2-(azidomethyl)oxazolo[5,4-*d*]pyrimidin-7(6*H*)-one (2c)**. Following the general procedure above for the preparation of compound **2b**, compound **6c** (0.25 g, 0.67 mmol) afforded a crude product that was purified by flash column chromatography (20% CH_3_OH in EtOAc) to give compound **2c** as a tan solid (47 mg, 34%). Mp > 250 °C. *R_f_* = 0.55 (20% CH_3_OH in EtOAc). ^1^H NMR (400 MHz, 0.5% NaOH in D_2_O) δ 4.42 (s, 2H). ^13^C NMR (100 MHz, 0.5% NaOH in D_2_O) δ 168.5, 166.9, 162.0, 153.9, 112.2, 49.0. IR (KBr) 3407, 2111, 2049, 1629, 1448, 1315, 1081 cm^-1^. MS (CI) m/z 208 (MH^+^). HRMS calcd for C_6_H_6_N_7_O_2_ (MH^+^) 208.0583, found 208.0587.

**5-Amino-2-methyloxazole-4-carboxylic acid ethyl ester (3a)** [1]. Anhydrous HCl was bubbled through a solution of ethyl acetamidocyanoacetate (0.14 g, 0.82 mmol) in dry THF (16 mL) for 1 h at room temperature. The reaction flask was capped, and the mixture was stirred at room temperature for an additional 3 h. Ether was added, and the precipitate was collected by filtration. The precipitate was washed with ether, and dissolved in CH_2_Cl_2_. Saturated aqueous NaHCO_3_ was added, and the pH of the aqueous layer was adjusted to 9 by the addition of sodium carbonate. The organic layer was separated, and the aqueous layer was extracted with CH_2_Cl_2_. The combined organic extracts were dried over MgSO_4_, and evaporated. The residue was purified by flash column chromatography (33% EtOAc in hexane) to give the title compound **3a** as a white solid (88 mg, 63%). R_f_ (1:5 MeOH/ CHCl_3_): 0.6; mp = 153-155 °C (lit. [1] 130-131 °C); ^1^H NMR (CD_3_OD, 300 MHz) δ 4.28 (q, 2H, J = 7 Hz), 2.29 (s, 3H), 1.33 (t, 3H, J = 7 Hz); ^13^C NMR (CD_3_OD, 75 MHz) δ 163.51, 160.91, 150.56, 102.21, 59.63, 13.77, 11.89; LRMS (CI) 171 (MH^+^); HRMS (CI) m/z 171.0760 (Calc. 171.0769, C_7_H_10_N_2_O_3_).

**5-Amino-2-isopropyloxazole-4-carboxylic acid ethyl ester (3b).** Following the procedure above for the preparation of compound **3a**, compound **5b** (0.22 g, 1.11 mmol) afforded a crude product that was purified by flash column chromatography (30% EtOAc in hexane) to give the title compound **3b** as a white powder (0.14 g, 64%). Mp = 109-111 °C. ^1^H NMR (400 MHz, CDCl_3_) δ 5.46 (br, 2H), 4.30 (q, *J* = 7.2 Hz, 2H), 2.95-2.90 (m, 1H), 1.34 (t, *J* = 7.2 Hz, 3H), 1.26 (d, *J* = 6.8 Hz, 6H). ^13^C NMR (100 MHz, CDCl_3_) δ 162.5, 158.9, 157.4, 104.2, 60.8, 29.5, 21.7, 16.1. IR (KBr) 3281, 3123, 2984, 1698, 1453, 1095 cm^-1^. MS (CI) m/z 199 (MH^+^). HRMS calcd for C_9_H_15_N_2_O_3_ (MH^+^) 199.1083, found 199.1080.

**5-Amino-2-(azidomethyl)oxazole-4-carboxylic acid ethyl ester (3c).** Following the general procedure above for the preparation of compound **3a**, compound **5c** (0.24 g, 1.1 mmol) afforded a crude product which was purified by flash column chromatography (33% EtOAc in hexanes) to give the title compound **3c** as a yellowish powder (0.15 g, 67%). Mp = 100-102 °C. *R_f_* = 0.45 (50% EtOAc in hexane). ^1^H NMR (300 MHz, CDCl_3_) δ 5.55 (br, 2H), 4.32 (q, *J* = 7.2 Hz, 2H), 4.28 (s, 2H), 1.35 (t, *J* = 7.2 Hz, 3H). ^13^C NMR (100 MHz, CDCl_3_) δ 163.3, 160.0, 147.1, 104.8, 60.4, 46.5, 14.5. IR (KBr) 2984, 2931, 2109, 1752, 1685, 1647, 1452, 1268, 1129 cm^-1^. MS (CI) m/z 212 (MH^+^). HRMS calcd for C_7_H_10_N_5_O_3_ (MH^+^) 212.0784, found 212.0780.

**Cyano(isobutyrylamino)acetic acid ethyl ester (5b)** [2]. To a suspension of Zn (1.84g, 28.0 mmol) in THF (10 mL) was added acetic acid (1.69 mL) dropwise and followed by isobutyric anhydride (1.72 mL). The mixture was stirred at 0 °C for 0.5 h until the solution became turbid. Oxime **4** (2.0 g, 14.0 mmol) was added slowly and the mixture was stirred at 0 °C for 0.5 h, and then allowed to warm to rt over 4 h. Saturated aqueous NaHCO_3_ was added to the reaction mixture until the pH was approximately 7. The aqueous layer was extracted with ethyl acetate, and the combined organic extracts dried over MgSO_4_, evaporated, and purified by column chromatography (EtOAc/Hexane = 1/1) to give a **5b** white solid (1.31 g, 47%). Mp = 129-131 °C (lit [2] 134 °C). ^1^H NMR (400 MHz, CDCl_3_) δ 6.60 (br, 1H), 5.52 (d, J = 7.6 Hz, 1H), 4.34 (q, *J* = 7.2 Hz, 2H), 2.53-2.47 (m, 1H), 1.35 (t, *J* = 7.2 Hz, 3H), 1.20 (d, *J* = 2.8 Hz, 3H), 1.18 (d, *J* = 3.2 Hz, 3H). ^13^C NMR (100 MHz, CDCl_3_) δ 175.5, 162.6, 114.0, 64.8, 43.8, 36.1, 20.5, 15.5. IR (KBr) 3292, 1740, 1653, 1533, 1312, 1214 cm^-1^. MS (CI) m/z 199 (MH^+^). HRMS calcd for C_9_H_15_N_2_O_3_ (MH^+^) 199.1083, found 199.1089.

**[(Azidoacetyl)amino]cyanoacetic acid ethyl ester (5c).** To 10 g of aluminum foil was added 100 mL of a 5% HgCl_2_ solution in H_2_O. After the HgCl_2_ had remained in contact with the foil for 10 min, the amalgamated foil was washed with three 500 mL portions of distilled water and a solution of 10 g oxime **4** (10 g, 0.07 mol) in MeOH (40 mL) and H_2_O (40 mL) was added at 0 °C. The reaction mixture was stirred for 4 h, and then filtered and the amalgamated foil washed with MeOH. The combined filtrates were evaporated to afford 7.4 g (81%) of crude aminocyanoacetate which was used without further purification. To a solution of this aminocyanoacetate (0.5 g, 3.9 mmol) in dry CH_2_Cl_2_ (10 mL) was added Et_3_N (1.09 mL, 7.8 mmol) at 0 °C. Azidoacetyl chloride [3] (0.47 g, 3.9 mmol) was added dropwise. The reaction mixture was stirred at 0 °C for 0.5 h, and allowed to warm to room temperature for another 3 h. The reaction mixture was quenched with saturated aqueous NH_4_Cl, and extracted with EtOAc. The organic layers were combined, dried over MgSO_4_, and evaporated. The crude product was purified by flash column chromatography (33% EtOAc in hexane) to give the title compound **5c** as a light-yellow powder (0.305 g, 37%). *R_f_* = 0.5 (50% EtOAc in hexane). ^1^H NMR (300 MHz, CDCl_3_) δ 7.42 (d, *J* = 9.0 Hz, 1H), 5.48 (d, *J* = 9.0, 1H), 4.33 (q, *J* = 7.0 Hz, 2H), 4.08 (s, 2H), 1.3 (t, *J* = 7.0 Hz, 3H). ^13^C NMR (75 MHz, CDCl_3_) δ 167.0, 162.8, 113.4, 64.4, 52.0, 42.7, 13.8. IR (KBr) 3330, 2925, 2887, 2099, 1752, 1693, 1518, 1285 cm^-1^. MS (CI) m/z 212 (MH^+^). HRMS calcd for C_7_H_10_N_5_O_3_ (MH^+^) 212.0784, found 212.0786.

**5-(3-Benzoyl-thioureido)-2-methyloxazole-4-carboxylic acid ethyl ester (6a).** A solution of 0.5 g (2.94 mmol) of compound **3a** and 0.475 mL (3.50 mmol) of benzoyl isothiocyanate in 25 mL of CH_3_CN was heated under reflux for 2h. The solvent was evaporated and the residue was dissolved in EtOAc and washed with water. The organic layer was dried over Na_2_SO_4_, the solvent evaporated, and the residue subjected to flash chromatography (50% EtOAc in hexane) to afford compound **6a** as a slightly yellow solid (500 mg, 51%). Mp = 130-132 °C. R_f_ (50% EtOAc in hexane): 0.5. ^1^H NMR (CDCl_3_, 300 MHz) δ 9.35 (s, 1H), 7.92 (d, 2H, J = 6.9 Hz), 7.67-7.52 (m, 3H), 4.44 (q, 2H, J = 6.9 Hz), 2.52 (s, 3H), 1.39 (t, 3H, J = 6.9 Hz); ^13^C NMR (CDCl_3_, 75 MHz) δ 176.38, 166.36, 161.82, 156.14, 148.72, 134.01, 130.88, 129.15, 127.74, 117.04, 61.43, 14.31, 14.05; IR (neat) 1754, 1674, 1636, 1520, 1292, 1218 cm^-1^; LRMS (CI) 334 (MH^+^); HRMS (CI) m/z 334.0858 (Calc. 334.0862, C_15_H_16_N_3_O_4_S).

**5-(3-Benzoyl-thioureido)-2-isopropyloxazole-4-carboxylic acid ethyl ester (6b).** To a solution of compound **3b** (0.5 g, 3.54 mmol) in dry CH_3_CN (20 mL) was added benzoyl isothiocyanate (0.72 mL, 5.31 mmol) at room temperature. The mixture was heated under reflux for 2 h. The reaction mixture was evaporated and the residue was subjected to flash column chromatography (25% EtOAc in hexane) to give the title compound **6b** as a light yellow solid (0.42 g, 40%). Mp = 105-106 °C. ^1^H NMR (400 MHz, CDCl_3_) δ 13.7 (s, 1H), 9.38 (s, 1H), 7.94-7.92 (m, 2H), 7.67-7.63 (m, 1H), 7.52-7.51 (m, 2H), 4.45 (q, *J* = 7.2 Hz, 2H), 3.17-3.10 (m, 1H), 1.40 (t, *J* = 7.2 Hz, 3H), 1.37 (d, J = 4.4 Hz, 6H). ^13^C NMR (100 MHz, CDCl_3_) δ 175.2, 165.4, 162.7, 161.1, 147.9, 133.6, 130.6, 128.8, 127.4, 116.6, 62.2, 29.8, 21.5, 15.9. IR (KBr) 3381, 1720, 1624, 1525, 1491, 1287 cm^-1^. MS (CI) m/z 361 (MH^+^). HRMS calcd for C_17_H_19_N_3_O_4_S (MH^+^) 361.1096, found 361.1090

**2-(Azidomethyl)-5-(3-benzoyl-thioureido)oxazole-4-carboxylic acid ethyl ester (6c).** To a solution of compound **3c** (25 mg, 0.12 mmol) in dry CH_3_CN (5 mL) was added benzoyl isothiocyanate (0.02 mL, 0.14 mmol) at room temperature. The mixture was heated under reflux for 3 h. The solvent was evaporated and the residue was subjected to flash column chromatography (25% EtOAc in hexane) to give the title compound **6c** as a yellowish solid (18 mg, 41%). Mp = 136-137.5 °C. *R_f_* = 0.8 (50% EtOAc in hexane). ^1^H NMR (300 MHz, CDCl_3_) δ 9.36 (br, 1H), 7.90 (d, *J* = 6.6 Hz, 2H), 7.62 (t, *J* = 6.6 Hz, 1H), 7.51 (t, *J* = 6.6 Hz, 2H), 4.52 (q, *J* = 7.0 Hz, 2H), 4.51 (s, 2H), 1.38 (t, *J* = 7.0 Hz, 3H). ^13^C NMR (75 MHz, CDCl_3_) δ 176.6, 166.8, 161.8, 153.1, 150.2, 134.4, 131.1, 129.5, 128.1, 117.4, 62.1, 46.9, 14.6. MS (CI) m/z 375 (MH^+^). HRMS calcd for C_15_H_15_N_6_O_4_S (MH^+^) 375.0876, found 375.0880.

**5-Mercapto-2-methyloxazolo[5,4-*d*]pyrimidin-7(6*H*)-one (7a).** To a solution of compound **6a** (0.28 g, 0.66 mmol) in ethanol/water (1:1, 10 mL) was added KOH (94.2 mg, 1.18 mmol). The mixture was heated to reflux for 2 h. The reaction mixture was cooled and acidified with 1N HCl. The precipitate was isolated by filtration and purified by C_18_ reverse phase chromatography (1:1 CH_3_OH/H_2_O) to give a white powder that was recrystallized from 50% CH_3_OH in acetone to afford a white solid (0.12 g, 76%). Mp 281-283 °C. *R_f_* = 0.25 (20% CH_3_OH in EtOAc). ^1^H NMR (300 MHz, D_2_O) δ 2.33. ^13^C NMR (75 MHz, D_2_O) δ 174.4, 165.7, 160.5, 159.8, 114.7, 13.5. IR (KBr) 3403, 1647, 1309 cm^-1^. MS (CI) m/z 184 (MH^+^). HRMS calcd for C_6_H_6_N_3_O_2_S (MH^+^) 184.0178, found 184.0181.

**2-Isopropyl-5-mercaptooxazolo[5,4-*d*]pyrimidin-7(6*H*)-one (7b).** Following the general procedure above for the preparation of compound **7a**, compound **6b** (55 mg, 0.15 mmol) afforded a precipitate that was purified by C_18_ reverse phase chromatography (1:1 CH_3_OH/H_2_O ) to give the title compound **7b** as a white powder (25 mg, 77%). Mp = 230-232.5 °C. ^1^H NMR (400 MHz, 0.5% NaOH in D_2_O) δ 3.03-2.96 (m, 1H), 1.17 (d, *J* = 2.4 Hz, 6H). ^13^C NMR (100 MHz, 0.5% NaOH in D_2_O) δ 172.9, 166.3, 164.2, 158.3, 114.2, 29.7, 20.3. IR (KBr) 2411, 1684, 1583, 1293 cm^-1^. MS (CI) m/z 212 (MH^+^). HRMS calcd for C_8_H_10_N_3_O_2_S (MH^+^) 212.0494, found 212.0500.

**2-Methyl-5-(methylthio)oxazolo[5,4-*d*]pyrimidin-7(6*H*)-one (8a).** To a solution of compound **7a** (120 mg, 0.66 mmol) in a THF and water mixture (1:1, 10 mL) was added dropwise CH_3_I (0.06 mL, 0.98 mmol). The reaction mixture was stirred at room temperature for 2 h. The solvent was evaporated to ~ 3 mL and the precipitate was collected by filtration, dissolved in aqueous NaOH (0.1%), and purified by C_18_ reverse phase chromatography (1:1 CH_3_OH/H_2_O) to give a white powder that was recrystallized from 50% CH_3_OH in acetone to afford the title compound as a white crystals (83 mg, 66%). Mp >300 °C. *R_f_* = 0.6 (20% CH_3_OH in EtOAc). ^1^H NMR (300 MHz, D_2_O) δ 2.28 (s, 3H), 2.22 (s, 3H). ^13^C NMR (75 MHz, D_2_O) δ 166.6, 166.5, 165.4, 159.1, 115.8, 13.7, 13.5. IR (KBr) 3357, 1612, 1453, 1300 cm^-1^. MS (CI) m/z 198 (MH^+^), 212 (MCH_3_^+^). HRMS calcd for C_7_H_8_N_3_O_2_S (MH^+^) 198.0337, found 198.0334.

**5-(Benzylthio)-2-methyloxazolo[5,4-*d*]pyrimidin-7(6*H*)-one (8b).** To a solution of compound **7a** (0.10 g, 0.55 mmol) in a THF and water mixture (1:1, 8 mL) was added benzyl bromide (0.11 g, 0.60 mmol). The reaction mixture was stirred at room temperature for 8 h. The reaction mixture was concentrated *in vacuo*, and the residue dissolved in aqueous NaOH (0.1%) and subjected to C_18_ reverse phase chromatography (CH_3_OH/H_2_O = 1/1) to give a white powder that was recrystallized from 50% CH_3_OH in acetone to afford the title compound **8b** as a white crystals (0.14 g, 71%). Mp = 218-219 °C. *R_f_* = 0.6 (20% CH_3_OH in EtOAc). ^1^H NMR (300 MHz, *d*-DMSO) δ 7.45 (d, *J* = 1.8 Hz, 2H), 7.42-7.26 (m, 3H), 4.44 (s, 2H), 2.51 (s, 3H). ^13^C NMR (75 MHz, *d*-DMSO) δ 163.4, 159.1, 158.7, 156.4, 136.6, 129.1, 128.5, 127.5, 117.4, 34.1, 14.0. IR (KBr) 2546, 1697, 1516, 1245 cm^-1^. MS (CI) m/z 274 (MH^+^). HRMS calcd for C_13_H_12_N_3_O_2_S (MH^+^) 274.0650, found 274.0654.

**2-Methyl-5-(prop-2-ynylsulfanyl)oxazolo[5,4-*d*]pyrimidin-7(6*H*)-one (8c).** To a solution of compound **7a** (0.10 g, 0.55 mmol) in a THF and water mixture (1:1, 8 mL) was added propargyl bromide (97 mg, 0.82 mmol). The reaction mixture was stirred at room temperature overnight. The precipitate was isolated by filtration, treated with dilute NH_4_OH, and purified by flash silica gel chromatography (4:1 EtOAc/CH_3_OH ) to give a white powder that was recrystallized from 50% CH_3_OH in acetone to afford the title compund **8c** as white crystals (70 mg, 58%). Mp = 233-236 °C. ^1^H NMR (400 MHz, 2:1 *d*-DMSO/CD_3_OD) δ 3.95 (d, *J* = 2.8 Hz, 2H), 2.94 (t, *J* = 2.8 Hz, 1H), 2.45 (s, 3H). ^13^C NMR (100 MHz, *d*-DMSO) δ 163.6, 159.2, 158.2, 156.7, 117.6, 79.5, 74.1, 19.1, 14.1. IR (KBr) 3423, 3267, 1685. 1591, 1337, 1255 cm^-1^. MS (CI) m/z 222 (MH^+^). HRMS calcd for C_9_H_8_N_3_O_2_S (MH^+^) 222.0337, found 222.0339.

**2-Methyl-5-[(3,4,5-trihydroxy-6-methoxytetrahydropyran-2-ylmethyl)sulfanyl]oxazolo[5,4-*d*]pyrimidin-7(6*H*)-one (8d).** To a solution of compound **7a** (0.10 g, 0.55 mmol) in a THF and water mixture (1:1, 8 mL) was added methyl 6-*O*-(tolylsulfonyl)-α-d-glucopyranoside [4] (0.23 g, 0.66 mmol). The reaction mixture was stirred at room temperature for 8 h. The precipitate was collected by filtration, treated with dilute NH_4_OH, and purified by flash silica gel chromatography (EtOAc/CH_3_OH = 4/1) to give a white powder that was recrystallized from 50% CH_3_OH in acetone to afford the title compound **8d** as white crystals (92 mg, 47%). Mp = 222-223 °C. ^1^H NMR (400 MHz, *d*-DMSO) δ 4.90 (br, 1H), 4.81 (br, 1H), 4.52 (d, *J* = 3.6 Hz, 1H), 4.77 (dd, *J* = 13.2, 2.8 Hz), 3.54 (ddd, *J* = 18.4, 9.2, 2.4 Hz, 1H), 3.40-3.37 (m, 1H), 3.23 (s, 3H), 3.24-3.20 (m, 1H), 3.20-3.15 (m, 1H), 3.03 (t, *J* = 9.2 Hz, 1H), 2.48 (s, 3H). ^13^C NMR (100 MHz, *d*-DMSO) δ 164.1, 160.4, 159.3, 157.2, 117.9, 100.5, 74.1, 73.7, 72.5, 70.6, 55.1, 33.6, 14.6. IR (KBr) 3401, 2947, 1723, 1582, 1536 cm^-1^. MS (CI) m/z 360 (MH^+^). HRMS calcd for C_13_H_18_N_3_O_7_S (MH^+^) 360.0865, found 360.0863.

**5-[(1-Benzyl-1*H*-1,2,3-triazol-4-ylmethyl)sulfanyl]-2-methyloxazolo[5,4-*d*]pyrimidin-7(6*H*)-one (9).** To a suspension of **8c** (97.1 mg, 0.44 mmol) and benzyl azide (62.2 mg, 0.44 mol) in a mixture of water and *tert*-butyl alcohol (1:1, 10 mL) was added copper (II) sulfate pentahydrate (5 mg, 23 μmol) followed by sodium ascorbate (4 mg, 23 μmol). The mixture was stirred at room temperature for 48 h. The mixture was cooled in ice, and the grey precipitate was collected by filtration. The crude product was purified by flash silica gel chromatography (4:1 EtOAc/CH_3_OH) to give a white powder that was recrystallized from 50% CH_3_OH in acetone to afford the title compound as white crystals (110 mg, 71%). Mp = 193-194 °C. ^1^H NMR (400 MHz, *d*-DMSO) δ 13.1 (br, 1H), 8.4 (br, 1H), 7.34-7.24 (m, 5H), 5.56 (s, 2H), 4.47 (s, 2H), 2.50 (s, 3H). ^13^C NMR (100 MHz, *d*-DMSO) δ 163.5, 159.9, 158.5, 156.6, 142.9, 135.9, 128.6, 128.1, 127.8, 123.9, 117.3, 52.8, 25.1, 13.9. IR (KBr) 1700, 1595, 1577, 1333, 1249, 1102 cm^-1^. MS (CI) m/z 355 (MH^+^). HRMS calcd for C_16_H_15_N_6_O_2_S (MH^+^) 355.0977, found 355.0979.

**2-Methyl-5-({1-[2-(morpholin-4-yl)ethyl]-1H-1,2,3-triazol-4-ylmethyl}sulfanyl)oxazolo[5,4-*d*]pyrimidin-7(6*H*)-one (10).** To a suspension of **8c** (69 mg, 0.31 mmol) and 2-morpholinoethyl azide (49 mg, 0.31 mol) in a mixture of water and *tert*-butyl alcohol (1:1, 10 mL) was added copper (II) sulfate pentahydrate (4 mg, 16 μmol) followed by sodium ascorbate (3 mg, 16 μmol). The mixture was stirred at room temperature for 48 h. The mixture was cooled in ice and the grey precipitate was collected by filtration. The crude product was purified by flash silica gel chromatography (4:1 EtOAc/CH_3_OH) to give a white powder that was recrystallized from CH_3_OH to afford the title compounds **10** as white crystals (66 mg, 63%). Mp = 212-213 °C. ^1^H NMR (400 MHz, *d*-DMSO) δ 8.00 (s, 1H), 4.43 (s, 2H), 4.40 (t, *J* = 5.2 Hz, 2H), 3.43 (t, *J* = 4.0 Hz, 4H), 2.67 (t, *J* = 5.2 Hz, 2H), 2.48 (s, 3H), 2.33 (t, *J* = 4.0 Hz, 4H). ^13^C NMR (100 MHz, *d*-DMSO) δ 164.1, 159.8, 159.2, 158.1, 143.0, 124.8, 117.7, 66.5, 57.6, 53.2, 47.0, 25.5, 14.3. IR (KBr) 2922, 2852, 2809, 1700, 1578, 1245, 1115 cm^-1^. MS (CI) m/z 378 (MH^+^). HRMS calcd for C_15_H_20_N_7_O_3_S (MH^+^) 378.1348, found 378.1353.

**RTA Expression and RTA/Inhibitor Activity Assay.**

Recombinant RTA was overexpressed in *E. coli* JM101 as described previously [5]. RTA activity and inhibition were determined by in vitro translation assay using Promega (Madison, Wisconsin) untreated rabbit reticulocyte lysate. 4 µL of the translation mix (1mM spermine tetrahydrochloride, 10 mM ATP, 2 mM GTP, 140 mM creatine phosphate, 1.25 M potassium acetate, 625 mM potassium chloride, 10 mg creatine kinase, 100 mM Tris/HCl, pH 7.6), 1.5 µL of 6.25 mM magnesium acetate, 1 µL of Promega amino acid minus methionine, 2 µL of 20 µCi/ul [^35^S]-methionine and 10 µL nuclease-free water, were added into 20 µL of lysate to initiate the translation process of endogenous globin mRNA. 10 µL of nuclease-free water was replaced with varying concentrations of RTA to assess potency. A fixed concentration of RTA (typically a RTA concentration that gives 90% protein synthesis inhibition) and varying concentrations of inhibitor candidates (0.05mM ~ 5mM) were added to assess inhibitor efficacy. Complete reaction mixtures were incubated at 30 °C for one hour. 10 µL of reaction sample were removed into 490 µL of the reaction stop solution (1 N NaOH/2% H_2_O_2_) with a 10 minutes incubation at 30 °C. At the end of the incubation, 900 µL of ice-cold 25% TCA was added to the reaction tube and incubate on ice for at least 5 minutes to precipitate the translation product. Finally, the translation product was collected by vacuum filtering, and the protein synthesis quantity was determined by [^35^S] incorporation obtained from the Perkin Elmer liquid scintillation analyzer. Since Promega rabbit reticulocyte lysate was more sensitive to RTA compared with previously used *Artemia salina* ribosome system^1^, 10 pM RTA (about 1nM in *Artemia salina* ribosome system ) with 90% protein synthesis inhibition was used to determine the compounds inhibitory effectiveness. Among all compounds tested, two of them, **2b** and **9**, showed RTA inhibition with IC_50_ of 2.8 mM and 1.6 mM, respectively.

**References**

1. Grifantini, M.; Stein, M. L. *Ann. Chim. (Rome, Italy)* **1965,** *55,* 576–582; *Chem. Abstr.* **1965,** *63,* 13234.
2. Golankiewicz, B.; Januszczyk, P.; Zeidler, J.; Popenda, M. *Nucleosides, Nucleotides Nucleic Acids* **2004,** *23,* 127–136. doi:[10.1081/NCN-120027822](http://dx.doi.org/10.1081/NCN-120027822)
3. Nicolaides, E. D.; Westland, R. D.; Wittle, E. L. *J. Am. Chem. Soc.* **1954,** *76,* 2887–2891. doi:[10.1021/ja01640a009](http://dx.doi.org/10.1021/ja01640a009)
4. Griffith, B. R.; Krepel, C.; Fu, X.; Blanchard, S.; Ahmed, A.; Edmiston, C. E.; Thorson, J. S. *J. Am. Chem. Soc.* **2007,** *129,* 8150–8155. doi:[10.1021/ja068602r](http://dx.doi.org/10.1021/ja068602r)
5. Ready, M. P.; Kim, Y.; Robertus, J. D. *Proteins: Struct., Funct., Genet.* **1991,** *10,* 270–278. doi:[10.1002/prot.340100311](http://dx.doi.org/10.1002/prot.340100311)
