# Supplementary material for: Synthesis of 2-substituted 9-oxa-guanines {5-aminooxazolo[5,4-d]pyrimidin-7(6H)-ones} and 9-oxa-2-thio-xanthines {5-mercaptooxazolo[5,4-d]pyrimidin-7(6H)-ones}
Source: Beilstein J Org Chem. 2008 Jul 25;4:26. doi: 10.3762/bjoc.4.26 (PMC2511024; doi:10.3762/bjoc.4.26)
Supplement: File 2 — Copies of 1H and 13C NMR spectra of compounds 2b,c; 3a–c; 5b,c; 6a–c; A; 7a,b; 8a–d; 9; and 10. [file Beilstein_J_Org_Chem-04-26-s002.doc]

**Supporting Information: ^1^H and ^13^C NMR Spectra**

**Synthesis of 2-substituted 9-oxa-guanines {5-aminooxazolo[5,4-*d*]pyrimidin-7(6*H*)-ones} and 9-oxa-2-thio-xanthines {5-mercaptooxazolo[5,4-*d*]pyrimidin-7(6*H*)-ones}**

Subrata Mandal^1^, Wen Tai Li^1^, Yan Bai^2^, Jon D. Robertus^2^ and Sean M. Kerwin*^,1,2^

Address: ^1^College of Pharmacy, 1 University Station, University of Texas, Austin, TX, 78712, USA and ^2^Department of Biochemistry,1 University Station, University of Texas, Austin, TX, 78712, USA

Email: Sean M. Kerwin - [skerwin@mail.utexas.edu](mailto:skerwin@mail.utexas.edu)

* Corresponding author
